# Primary TKA Patients with Quantifiably Balanced Soft-Tissue Achieve Significant Clinical Gains Sooner than Unbalanced Patients

**DOI:** 10.1155/2014/628695

**Published:** 2014-08-18

**Authors:** Kenneth A. Gustke, Gregory J. Golladay, Martin W. Roche, Leah C. Elson, Christopher R. Anderson

**Affiliations:** ^1^Florida Orthopaedic Institute, 13020 North Telecom Parkway, Temple Terrace, FL 33637, USA; ^2^Department of Orthopaedic Surgery, VCU Medical Center, P.O. Box 980153, Richmond, VA 23298-0153, USA; ^3^Department of Orthopedic Surgery, Holy Cross Hospital, 5597 North Dixie Highway, Fort Lauderdale, FL 33334, USA; ^4^Department of Clinical and Bioengineering Research, Orthosensor Inc., 1855 Griffin Road, Suite A-310, Dania, FL 33004, USA

## Abstract

Although total knee arthroplasty has a high success rate, poor outcomes and early revision are associated with ligament imbalance. This multicenter evaluation was performed in order to provide 1-year followup of a previously reported group of patients who had sensor-assisted TKA, comparing the clinical outcomes of quantitatively balanced versus unbalanced patients. At 1 year, the balanced cohort scored 179.3 and 10.4 in KSS and WOMAC, respectively; the unbalanced cohort scored 156.1 and 17.9 in KSS and WOMAC (*P* < 0.001; *P* = 0.085). The average activity level scores of quantitatively balanced patients were 68.6 (corresponding to tennis, light jogging, and heavy yard work), while the average activity level of unbalanced patients was 46.7 (corresponding to light housework, and limited walking distances) (*P* = 0.015). Out of all confounding variables, a balanced articulation was the most significant contributing factor to improved postoperative outcomes (*P* < 0.001).

## 1. Introduction

Advances in implant technology and surgical technique have contributed to the long withstanding success of total knee arthroplasty (TKA), such that the procedural survivorship is now as high as 95.6% at 10 years [1]. Partly as a result of its success, TKA is currently the most frequently performed orthopaedic intervention in the United States [2], the volume of which is projected to reach over 2.5 million procedures, annually, by 2030 [3].

While TKA routinely restores function for the majority of patients, the incidence of early- and late-stage revisions is nonetheless increasing. The cost associated with this revision burden is projected to reach a staggering 13 billion dollars, annually [1, 4, 5]. Complications reported by post-TKA patients include pain (44%, multilocational), sensation of instability (21% reason for revision), and joint stiffness (17% reason for revision), which are problems that may be attributed to soft-tissue imbalance [2, 6, 7]. One of the possible reasons for the substantial prevalence of such complications is the traditional subjectivity associated with defining soft-tissue balance [8].

As the projected annual number of primary TKA procedures continues to rise [3], it has become imperative that a priority be placed on developing new methods to avoid costly postoperative complications. One such method includes the integration of intraoperative sensing technology into the surgical workflow, allowing surgeons to dynamically quantify* in vivo* kinetics associated with soft-tissue balancing. These sensors provide objective digital feedback to the surgeon, during the procedure, augmenting the traditional tactile methods used for balancing [3].

The early postoperative outcomes of a prospective group of patients that have undergone sensor-assisted TKA are promising [9]. However, previously reported literature is limited to a 6-month followup window; longer followup is thereby integral to further exploring the efficacy of clinical gains after sensor-assisted TKA.

Thus, the purpose of this evaluation is to report on the disparity between the patient-reported outcomes scores of quantitatively balanced versus unbalanced patients, at 1 year, using a group of 135 multicenter patients who have been previously reported in the literature [9].

## 2. Patients/Methods

### 2.1. Patients and Device

A cohort of 135 prospective patients have had primary TKA performed with the use of the VERASENSE Knee System (OrthoSensor Inc., Dania Beach, FL), which was used in conjunction with the Triathlon Knee System (Stryker, Mahwah, NJ). This cohort represents the contribution of eight surgeons from eight sites in the United States. All patients included in the following analysis have been previously reported in the literature [9] and have since been evaluated at the one-year followup interval.

Patients were eligible for enrollment in this study if they were a candidate for primary TKA, over the age of 50, with a diagnosis of osteoarthritis, avascular necrosis, rheumatoid or other inflammatory arthritis, or posttraumatic arthritis. Exclusion criteria included ligament insufficiencies, prior surgeries, prior TKA, posterior lateral reconstructions, osteotomies, or tibia plateau fractures.

Since the publication of early clinical data on the original 176 patients [9], 22 were lost to followup (for varying reasons unrelated to their outcomes, i.e., patient relocating and disinterest in continuing to be seen annually), 7 have yet to be seen for their 1-year clinical visit, and 12 have pending clinical visits due to a practice relocation by their respective surgeon. No revisions have been reported in this study. Thus, 135 patients have had 1-year followup evaluations and are included in the analysis herein.

Preoperatively, comorbidities were captured in order to account for any possible confounding outcomes variables. For all patients, at all intervals, the following kinematic data was captured: anatomic alignment and ROM. Also at each clinical followup visit, activity levels and two patient-reported outcomes measures were administered, including the American Knee Society Score (KSS) and the Western Ontario and McMaster Universities Osteoarthritis Index (WOMAC) [10, 11]. Activity level was based on a 5-level scale: “bedridden” being the category of least amount of ambulation and “heavy labor” being the category of greatest amount of ambulation. The full scale includes the following physical states (in ascending order of increasing ambulation): “bedridden,” “sedentary,” “semisedentary,” “light labor,” “moderate labor,” and “heavy labor.” Patients were provided with several examples of the kind of activities that one might expect to be able to perform in each category without pain or inhibition and were asked to select which category is most representative of their joint performance. For instance, “semisedentary” patients would be able to perform light household cleaning, white collar office work, or benchwork; “heavy labor” patients would be able to perform vigorous sports and/or lift 50–100 lbs. For the purposes of statistical analysis only, each descriptive category was designated with a numeric representation, at 20-point intervals, beginning at 0 as follows: “bedridden” is 0, “sedentary” is 20, “semisedentary” is 40, “light labor” is 60, “moderate labor” is 80, and “heavy labor” is 100.

### 2.2. Surgical Protocol

All procedures were performed through a medial parapatellar, subvastus, or midvastus approach, according to individual surgeon preference. Standard bony cuts were made with or without the use of navigation, at the discretion of each surgeon.

Once the femoral and tibial trial components had been placed, a polyethylene trial was inserted in order to assess appropriate size and joint stability. Once appropriate tibial insert size was determined, the corresponding VERASENSE sensor was activated, registration was verified, and the VERASENSE sensor was inserted. Custom shims were affixed to the sensor undersurface to replicate the thickness of the standard trial, as necessary.

With the sensor in place, tibial tray rotation was visually quantified using the contact point location of the tibiofemoral interface, as shown by the sensor system. The mid- to medial-third of the tibial tubercle was used as an anatomical reference to set initial tibial tray rotation. If rotational correction was required, a pin was placed in either an anteromedial or an anterolateral position to stabilize any translational motion during rotational correction. Correction for rotational incongruency was shown as symmetrical tibiofemoral contact point location, with the knee in full extension, as shown by the sensor system.

Once appropriate tibial tray rotation was achieved, soft-tissue balance was evaluated with the joint in three positions: full extension (0–10 degrees), midflexion (45 degrees), and in 90 degrees of flexion. Varus/Valgus stress testing was performed in extension and in 10 and 45 degrees of flexion. With the capsule closed, medial and lateral load measurements and center of load were documented at 10, 45, and 90 degrees of flexion. During the evaluation of mediolateral loading, it was important that no axial compression was applied across the joint. PCL stability assessed with a posterior drawer test was applied at 90 degrees of flexion with the hip in neutral rotation. Flexion balance was achieved when femoral contact point position was within the midposterior third of the tibial insert, intercompartmental loads were balanced, central contact points displayed less than 10 mm of excursion across the bearing surface during a posterior drawer test, and appropriate rollback was seen through ROM. A tight flexion gap during surgery was displayed by the sensor system as excessive pressures with combined femoral contact position in the posterior compartments. This was corrected through gradual release of the PCL, or in some instances with the addition of tibial slope. Excessive excursion of the femoral contact points which exhibited translation through the midanterior thirds of the tibial trial was indicative of laxity in the PCL. Surgical correction of PCL laxity required a thicker tibial insert, anterior-constrained insert, or a posterior-stabilized knee design. “Pie crusting” ligament releases were performed, as necessary, until the desired balance was achieved.

Pie crusting, as described by Bellemans et al., was applied for selective medial or lateral soft-tissue balancing [12]. Using a 19-gauge needle or number 11 blade (Figure 4), multiple punctures were made perpendicular to the fibers of the MCL or lateral ligaments to incrementally lengthen the ligament until the intercompartmental pressures were deemed acceptable by the individual surgeon. This technique is performed gradually, with the surgeon cycling the knee through flexion and extension after several punctures to allow the ligament to lengthen. All surgeons documented all intraoperative soft-tissue releases performed. Final load measurements and contact point location were recorded prior to cementing the final components.

### 2.3. Definition of “Balance” and Statistics

**Figure 1:**
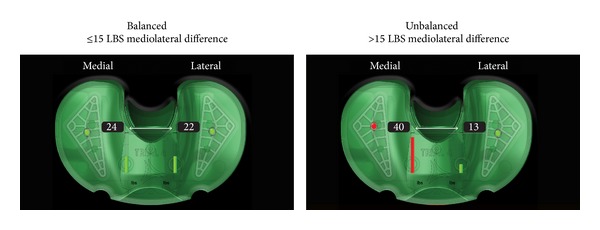
Coronal plane “balance” was defined as a mediolateral intercompartmental loading difference of ≤15 pounds; all transtibial loading exceeding 15 pounds of mediolateral intercompartmental difference was classified as “unbalanced.”

For the purpose of this evaluation, quantitative “balance” was defined as a mediolateral intercompartmental loading difference of ≤15 pounds; all transtibial loading exceeding 15 pounds of mediolateral intercompartmental difference was classified as “unbalanced” (Figure 1). The balancing standards must have necessarily been maintained through the range of motion, based on quantitative measurement in extension (10 degrees), in midflexion (45 degrees), and at 90 degrees of flexion. These loading values were selected based on (1) biomechanical research conducted to elucidate condylar contact pressures while the joint is in a passive state [13], (2) intraoperative observations made by experienced surgeons that quantified 2 mm of opening with Varus/Valgus stress and load changes coupled with navigation, and (3) the observation of significant declination in postoperative outcomes scores in patients with an intercompartmental loading difference which exceeded 20 lbs. [9].

Analysis of the data was performed using SPSS version 21 (SPSS Inc., Chicago, IL). Comparative statistics were executed to contrast the outcomes data at 1 year. These data were stratified by two groups: those with a “balanced” joint and those with an “unbalanced” joint. An observed power analysis was performed in order to determine if there was statistical strength in comparing the two groups, preset with a minimum power of 0.8, *α* = 0.05. The results of the analysis met the minimum criteria necessary to statistically compare the balanced and unbalanced groups (*P* = 0.005).

Analysis of variance (ANOVA) was used to assess any outcomes differences between the two groups, with post hoc *t*-tests to demonstrate significance. A multivariate logistic regression was performed to assess and extrapolate any confounding variable influence on outcomes scores. Significance for all analyses was defined as a *P* value <0.05.

## 3. Results

### 3.1. Descriptive Analysis: KSS, WOMAC, and Activity Level

Of the 135 patients with sensor-assisted surgery, 13% remained unbalanced by surgeon discretion. “Surgeon discretion,” in this analysis, indicates that the surgeon recognized and accepted the “unbalanced” intercompartmental load difference as presented by the VERASENSE system but felt that the knee was in a clinically acceptable state. Due to the novelty of this study—having no precedent—the multicenter evaluation was initiated for observational purposes, guided by data extracted from biomechanical analyses, as mentioned previously. The majority of these unbalanced patients presented early in the enrollment period, which is consistent with the learning curve that can typically be expected with the implementation of new technology [14]. In the case of using the intraoperative sensors, it was not immediately clear if the 15 lb threshold for balance would be clinically relevant. Thus, surgeons corrected the joints to the extent that they felt was appropriate, based on their training. However, as data became available, early postoperative outcomes indicated that the quantitative loading threshold was associated with higher and more favorable patient-reported outcomes scores. At that point, the surgeons in this study began to actively correct to the balanced threshold. Therefore, the initial “learning curve” of this evaluation was with respect to observing patients and discovering how to best clinically utilize sensor output. It should also be noted that no adverse anatomic abnormalities were present in the unbalanced group.

ANOVA analyses were executed to compare the mean values of demographic data between the two groups; no significant difference was found for any of the following variables. The mean age at surgery for the unbalanced cohort was 72 ± 7 years; mean age at surgery for the balanced cohort was 69 ± 8 years. The average BMI for the unbalanced group was 31 ± 6.4; the average BMI for the balanced group was 30 ± 5.3. The average female-to-male ratio for both groups was approximately 2 : 1.

Preoperatively, as per ANOVA analyses, there was also no statistical difference in alignment, ROM, or outcomes measures between the two groups. The preoperative alignment/ROM were 5.1/109° and 4.9/111° for the unbalanced and balanced groups, respectively. The average preoperative outcomes scores were comparable between the two groups: total KSS = 105 ± 24.6 and total WOMAC = 47 ± 14.8 [9]. Comorbidities were also captured preoperatively, including back/disc disease, cancer, cardiovascular disease (nonspecific), diabetes, endocrine disorder (nonspecific), gastrointestinal disorder (nonspecific), hypertension, and respiratory disorder (nonspecific). The proportion of patients exhibiting comorbidities was 66.2%. The outcomes of patients with comorbidities, when compared with patients who exhibited no comorbidities, exhibited no significant difference (*P* = 0.157, *P* = 0.342, *P* = 0.216 for KSS, WOMAC, and activity level, resp.).

**Figure 2:**
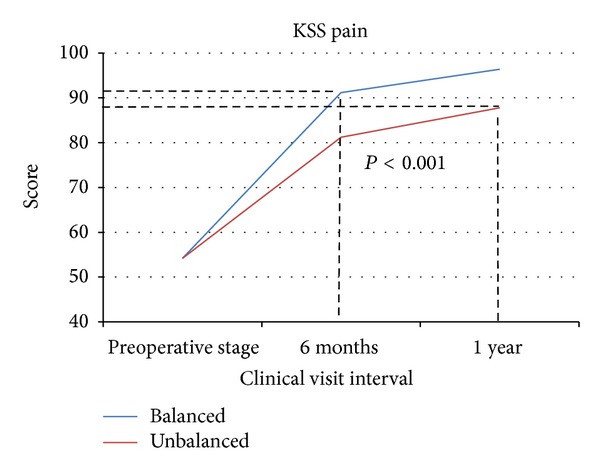
The lines represent the KSS pain scores for the balanced and unbalanced patients from preoperative stage to 1 year. The dashed lines indicate the disparity between the balanced and unbalanced patients' pain scores.

**Figure 3:**
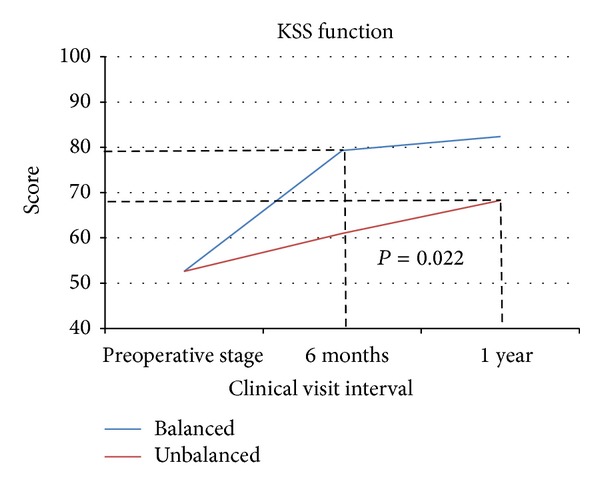
The lines represent the KSS function scores for the balanced and unbalanced patients from preoperative stage to 1 year. The dashed lines indicate the disparity between the balanced and unbalanced patients' functions scores.

At one year postoperatively, the average total KSS score of balanced patients exceeded that of unbalanced patients by 23.3 points (*P* < 0.001), 179 ± 17.2 and 156 ± 23.4 for the balanced and unbalanced groups, respectively. The balanced group average score for KSS pain and function was 96.4 and 82.4, respectively; the unbalanced group scored 87.8 and 68.3 points for pain and function (Figures 2 and 3). The disparity between the balanced and unbalanced patients' pain and function scores was also highly statistically significant (*P* < 0.001, *P* = 0.022).

**Figure 4:**
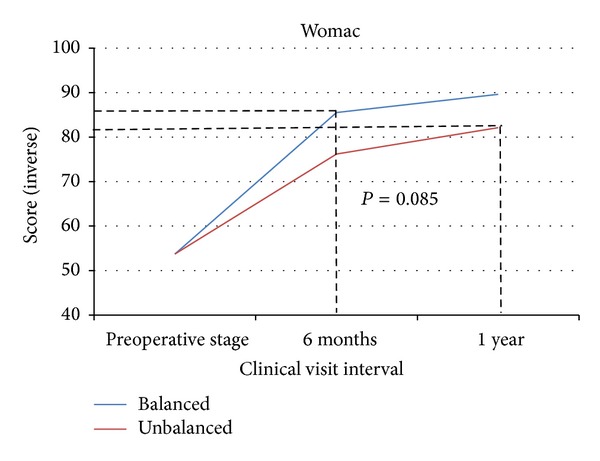
The lines represent the total WOMAC scores for the balanced and unbalanced patients from preoperative stage to 1 year. The dashed lines indicate the disparity between the balanced and unbalanced patients' total WOMAC scores.

The balanced group averaged 8 points more improvement in WOMAC scores than the unbalanced group (10 ± 11.8 and 18 ± 17 for balanced and unbalanced patients, resp.) (Figure 4). It is important to note that the WOMAC is scored with an inverse scale; lower scores indicate more improvement. While this difference did not prove to be statistically significant by the standards set forth for this analysis (*P* = 0.085), the authors believe that this is due, in part, to the large standard deviations associated with both cohorts.

**Figure 5:**
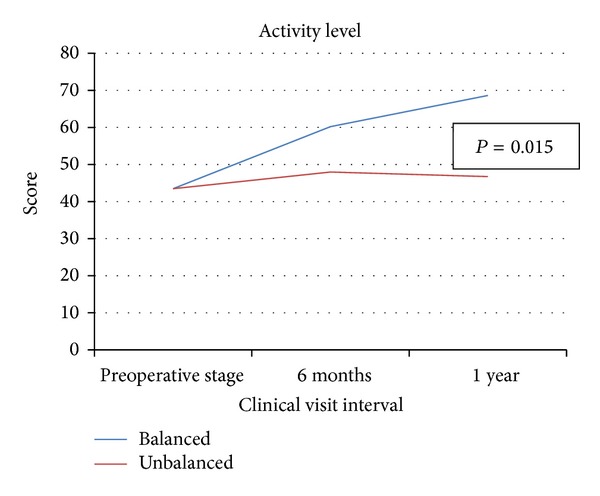
The lines indicate the change in patient activity levels for the balanced and unbalanced cohorts from preoperative stage to 1 year.

The 1-year postoperative activity levels between the two groups were also distinctly different (Figure 5). The balanced group's average activity level score was 68.6, which corresponds to the light to moderate labor categories (tennis, light jogging, and heavy yard work). The unbalanced patient's average activity level score was 46.7, which corresponds to the semisedentary range (light housework and walking for limited distances). The difference between the average scores was statistically significant (*P* = 0.015).

The most notable aspect of every outcomes measure collected is that the unbalanced patient scores at one year still failed to achieve the level of improvement of the balanced patient scores at 6 months. The dashed lines in Figures 2–5 visually indicate these limits to the unbalanced patient cohort improvement.

### 3.2. Statistical Analysis

A multivariate logistic regression analysis was performed for both KSS and WOMAC scores at the 1-year followup interval. The KSS and WOMAC scores, for all patients, represented the dependent end-point variables. All confounding variables that could have contributed to the observed scores of the outcomes measures were evaluated as independent. Those variables included BMI, gender, age at surgery, preoperative ROM, preoperative alignment, change in activity level (preoperative stage to 1 year), and joint state (“balanced” versus “unbalanced”). These variables are evaluated separately, and with every other possible combination of covariables, in order to assess which contributed most significantly to the scores observed. A step-wise multivariate logistic regression analysis was calculated to be the best-fit model for KSS and WOMAC (*P* < 0.001).

The regression model revealed that the variable exhibiting the most significant effect of improvement on KSS and WOMAC score was balanced joint state (*P* = 0.001; *P* = 0.014). This variable also represented the only significant variable when combined with all other possible confounders. Joint state was the most highly significant variable when analyzed independently, as well as with every other possible combination of variables included in the model (*P* = 0.001). Interestingly, there was also a concurrent significance observed with activity level (*P* = 0.022). However, activity level was not significant on its own. This leads to the belief that a balanced joint state may result in a higher activity level, the latter being dependent on the former.

## 4. Discussion

Total knee arthroplasty is consistently reported to successfully return function to patients suffering from late-stage osteoarthritis [15]. However, there is still a substantial proportion of TKA recipients who return to followup visits reporting pain, instability, and overall dissatisfaction [2, 6, 7]. Postoperative complications such as these have been attributed, in part, to soft-tissue imbalance. The prevalence of these complications may be due to the subjective nature with which ligament tension is evaluated [8]. In order to mitigate the continuation of soft-tissue based complications, microelectronics embedded in the tibial trial insert have been developed to quantify balance intraoperatively.

In this evaluation, the efficacy of using intraoperative sensing technology to verify ligament balance was assessed. While this group of patients had been previously reported in the literature [9], longer clinical followup was necessary to clearly define the full extent to which balanced patients clinically surpass unbalanced patients.

No significant difference was found between the balanced and unbalanced groups with respect to demographics, preoperative surgical variables (i.e., ROM and alignment), or preoperative outcomes scores. Furthermore, there was no significant difference in the outcomes of patients with concurrent comorbidities versus those without comorbidities. These values indicate that the balanced/unbalanced patients groups exhibited no preoperative bias which would have contributed to the difference observed in postoperative outcomes scores.

At 1 year postoperatively, the KSS, WOMAC, and activity level scores of balanced patients surpassed those of the unbalanced patients by 23.2, 8, and 22 points, respectively (*P* = 0.001, *P* = 0.085, and *P* = 0.015). The most notable trend of these three patient-reported outcomes measures is the discrepancies between 6-month and 1-year data between the two groups. The improvements made by unbalanced patients, at one year, still have not reached the improvement that balanced patients achieved by the 6-month followup interval (Figures 2–5). This suggests that verifiably balanced patients not only obtain statistically significant improvement in both pain and function levels versus unbalanced patients, but also do so in a shorter amount of time than their unbalanced counterparts. This trend could only be confirmed with the inclusion of 1-year data.

The 1-year regression analysis is in agreement with the same analysis executed for the 6-month data [9]: it suggests that the state of balance within each joint was the most significant factor contributing to the difference in patient-reported outcomes between the balanced and unbalanced groups. This finding is supported by the nonsignificance observed in preoperative outcomes measures and patient-specific variables.

The limitations to this study were as follows. (1) Only 13% of the group of sensor-guided TKA patients were classified as “unbalanced.” While this number represents enough statistical power to make cross-comparisons between quantitatively balanced and unbalanced patients (power (0.81); *α* = 0.05; *P* = 0.005), it is always preferable to have the largest cohorts possible. (2) There is no blinded study currently available to compare the outcomes of patients who have had sensor-guided TKA with those who have not. Thus some of the improvement in outcomes scores could have been attributed to the placebo effect of patients knowing that they were receiving technologically assisted TKA. However, because* both* groups were consented and informed of the nature of the sensor, it is unlikely that any bias in the quantitatively balanced group would be observed. (3) The true improvement of the quantitatively balanced patients is unlikely to be known with the current outcomes scores available. Many patients in the balanced cohort reached the maximum scores possible in both KSS and WOMAC, as early as 6 months, whereas the unbalanced patients did not for either scoring system. This creates a ceiling effect in our data, which may mean that the averages for the quantitatively balanced cohort are actually higher than can be effectively reported. (4) None of the eight participating surgeons were Triathlon Knee System users prior to initiating this study. Thus, the scores reported in this study may exhibit a trend towards deflation due to any learning curve that may have existed in the earliest patients. (5) This study was not randomized for enrollment. The original purpose of the multicenter study was for observational purposes only. Fortunately, there was enough statistical power and compliance of followup to make generalized statements regarding data comparison between the two groups. Future double-blinded, randomized studies will be conducted in similar groups of patients.

Evidence from this evaluation suggests that sensor-guided, quantifiably balanced TKA patients are statistically more likely to achieve reduced pain, improved function, and greater activity levels* sooner* than unbalanced patients. These findings may help to provide valuable insight into technological alternatives to soft-tissue balance and their potential for mitigating postoperative complications.
